# Global diversity and inferred ecophysiology of microorganisms with the potential for dissimilatory sulfate/sulfite reduction

**DOI:** 10.1093/femsre/fuad058

**Published:** 2023-10-05

**Authors:** Muhe Diao, Stefan Dyksma, Elif Koeksoy, David Kamanda Ngugi, Karthik Anantharaman, Alexander Loy, Michael Pester

**Affiliations:** Department of Microorganisms, Leibniz Institute DSMZ - German Collection of Microorganisms and Cell Cultures GmbH, Braunschweig D-38124, Germany; Department of Microorganisms, Leibniz Institute DSMZ - German Collection of Microorganisms and Cell Cultures GmbH, Braunschweig D-38124, Germany; Department of Microorganisms, Leibniz Institute DSMZ - German Collection of Microorganisms and Cell Cultures GmbH, Braunschweig D-38124, Germany; Department of Microorganisms, Leibniz Institute DSMZ - German Collection of Microorganisms and Cell Cultures GmbH, Braunschweig D-38124, Germany; Department of Bacteriology, University of Wisconsin-Madison, Madison, WI, 53706, USA; Division of Microbial Ecology, Centre for Microbiology and Environmental Systems Science, University of Vienna, Vienna A-1030, Austria; Department of Microorganisms, Leibniz Institute DSMZ - German Collection of Microorganisms and Cell Cultures GmbH, Braunschweig D-38124, Germany; Technical University of Braunschweig, Institute of Microbiology, Braunschweig D-38106, Germany

**Keywords:** sulfate reduction, sulfur oxidation, sulfur cycle, dissimilatory sulfite reductase, metagenomics, *dsrAB*

## Abstract

Sulfate/sulfite-reducing microorganisms (SRM) are ubiquitous in nature, driving the global sulfur cycle. A hallmark of SRM is the dissimilatory sulfite reductase encoded by the genes *dsrAB*. Based on analysis of 950 mainly metagenome-derived *dsrAB*-carrying genomes, we redefine the global diversity of microorganisms with the potential for dissimilatory sulfate/sulfite reduction and uncover genetic repertoires that challenge earlier generalizations regarding their mode of energy metabolism. We show: (i) 19 out of 23 bacterial and 2 out of 4 archaeal phyla harbor uncharacterized SRM, (ii) four phyla including the *Desulfobacterota* harbor microorganisms with the genetic potential to switch between sulfate/sulfite reduction and sulfur oxidation, and (iii) the combination as well as presence/absence of different *dsrAB-*types*, dsrL*-types and *dsrD* provides guidance on the inferred direction of dissimilatory sulfur metabolism. We further provide an updated *dsrAB* database including > 60% taxonomically resolved, uncultured family-level lineages and recommendations on existing *dsrAB*-targeted primers for environmental surveys. Our work summarizes insights into the inferred ecophysiology of newly discovered SRM, puts SRM diversity into context of the major recent changes in bacterial and archaeal taxonomy, and provides an up-to-date framework to study SRM in a global context.

## Teaser

Sulfate/sulfite reducing microorganisms are shaping Earth's interconnected sulfur and carbon cycles since the Archaean: this legacy unfolds in 27 archaeal and bacterial phyla encountered in diverse marine, terrestrial, and deep-subsurface environments.

## Introduction

The sulfur cycle is one of the most important biogeochemical cycles on Earth (Canfield and Farquhar 2012) tightly interacting with carbon, nitrogen, and metal cycling (Jørgensen 2021). It is mainly regulated by activities of sulfate/sulfite-reducing microorganisms (SRM) and sulfur-oxidizing microorganisms (SOM) as their counterparts (Dahl et al. 2008, Rabus et al. 2013, Rabus et al. 2015, Wasmund et al. 2017, Jørgensen 2021), which cycle sulfur between its most oxidized (sulfate, +VI) and its most reduced state (sulfide, -II). On a global scale, sulfate reduction is one of the dominant processes driving the mineralization of organic matter in anoxic environments. Of the estimated 260 Tmol C_org_ reaching the global seabed each year, one third is mineralized through sulfate reduction in marine sediments (Jørgensen 2021). About 90% of the end product, sulfide, is re-oxidized to sulfate either directly or indirectly at the expense of oxygen (Jørgensen 2021). This represents 25% of global oxygen consumption in sediments and has a direct impact on the redox state of Earth's surface. The relevance of sulfur cycling increases further in coastal sediments, where sulfate reduction accounts for 50% of C_org_ mineralization and re-oxidation of sulfide consumes 50% of the oxygen entering the sediment (Jørgensen 2021). In addition to marine sediments, marine oxygen minimum zones represent environments of active sulfur cycling. In these oxygen-depleted waters, sulfide produced by sulfate reduction is rapidly re-oxidized to sulfate by sulfide oxidation coupled to nitrate reduction (Canfield et al. 2010, Johnston et al 2014, Callbeck et al. 2018, van Vliet et al. 2021). Here, the term “cryptic sulfur cycle” was coined for the first time—“cryptic” because it was not evident from the spatial concentration profiles of inorganic sulfur compounds, in particular sulfide (Canfield et al. 2010).

While the importance of sulfate reduction in marine environments is well explained by the high availability of sulfate (ca. 28 mM), its role in biogeochemical cycling of anoxic freshwater environments such as lake sediments, groundwater, peatlands, or rice paddy fields is less obvious because of the low prevailing sulfate concentrations (typically 10–300 µM) (Pester et al. 2012). Nevertheless, the rates at which sulfate reduction proceeds can be equally high in marine and freshwater settings, resulting in rapid cycling of sulfur species in anoxic freshwater environments. Because of its less obvious relevance and high variability in space and time, the sulfur cycle in freshwater systems is often referred to as a cryptic or hidden sulfur cycle as well (Pester et al. 2012). The contribution of sulfate reduction to C_org_ mineralization in anoxic freshwater environments has not been evaluated so systematically as in marine environments, but single studies report values of 17–35% in lake sediments (Urban et al. 1994, Thomsen et al. 2004) and 36–50% in peatlands (reviewed in Pester et al. 2012). Yet another low-sulfate environment with cryptic sulfur cycling are deep marine subsurface sediments below the sulfate-methane transition zone. Here, sulfur cycling operates at very slow sulfate reduction rates. These slow rates are maintained by the re-supply of sulfate mediated by Fe(III)-driven sulfide oxidation (Holmkvist et al. 2011a,b, Pellerin et al. 2018, Jørgensen et al. 2019, Findlay et al. 2020).

Besides their relevance for biogeochemical cycling, SRM represent an important symbiotic guild in the mammalian intestinal tract (Barton et al. 2017) and are also beneficial in bioremediation, such as degrading hydrocarbons and removing heavy metals from sulfate-containing groundwater and wastewater (Muyzer and Stams 2008, Qian et al. 2019). However, they can also be an economic burden by causing steel corrosion or oil souring (Muyzer and Stams 2008, Rey et al. 2013, Rabus et al. 2015, Singh and Lin 2015, Wolf et al. 2022). In the context of climate change and human-induced eutrophication, it is noteworthy that oxygen concentrations in pelagic zones of the global ocean, coastal waters, and lakes have been declining for decades (Jenny et al. 2016, Breitburg et al. 2018). The resulting oxygen-deficient zones can turn euxinic (anoxic conditions with > 0.1 μM sulfide) upon release of toxic sulfide by SRM, which further aggravates the negative effects of oxygen shortage causing death to fauna including economically relevant fish, shrimp and crabs (Diaz and Rosenberg 2008, Jenny et al. 2016, Bush et al. 2017, Diao et al. 2018, van Vliet et al. 2021). On the other hand, SRM can also exert positive climate change effects. Especially in low-sulfate habitats with active cryptic sulfur cycling, such as rice paddy fields, peatlands and lake sediments, SRM compete for substrates with microorganisms involved in the methanogenic degradation network (Pester et al. 2012, Wörner et al. 2016, Wörner and Pester 2019). This leads to a partial diversion of the carbon flux from CH_4_ to CO_2_, which is the less potent greenhouse gas on a per molecule basis (Pester et al. 2012). Stimulation of cryptic sulfur cycling, e.g. by the addition or intrinsic activity of sulfide-oxidizing cable bacteria can thus exert positive effects on mitigation of methane emissions (Sandfeld et al. 2020, Scholz et al. 2020) or delay the development of euxinia (Seitaj et al. 2015).

**Figure 1.**
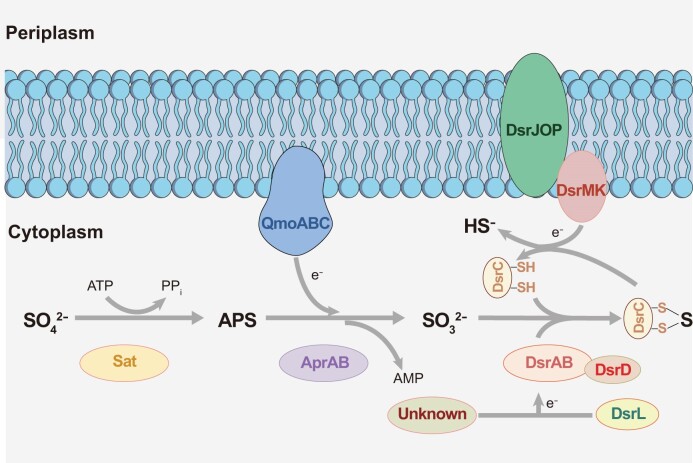
The pathway of dissimilatory sulfate reduction. The Dsr-pathway includes the enzymes sulfate adenylyltransferase (Sat), adenylyl phosphosulfate reductase (AprAB), dissimilatory sulfite reductase (DsrAB), and the sulfide releasing DsrC protein. The complexes QmoAB**(C)** and DsrMK(JOP) complement the pathway by transferring reducing equivalents towards AprAB and DsrC, respectively (Pereira et al. 2011, Ramos et al. 2012, Santos et al. 2015). Reducing equivalents required by DsrAB can be delivered by a yet unknown oxidoreductase or DsrL (Löffler et al. 2020). DsrD acts as an allosteric activator of DsrAB in sulfate/sulfite-, thiosulfate-, and organosulfonate reducers as well as sulfur disproportionating microorganisms (Ferreira et al. 2022).

Most SRM share a canonical core enzyme repertoire for carrying out dissimilatory sulfate reduction (Fig. 1). This intracellular pathway includes the enzymes sulfate adenylyltransferase (Sat), adenylyl phosphosulfate reductase (AprAB), dissimilatory (bi)sulfite reductase (DsrAB), and the sulfide releasing DsrC. The complexes QmoAB(C) and DsrMK(JOP) complement the pathway by transferring reducing equivalents towards AprAB and DsrC, respectively (Pereira et al. 2011, Ramos et al. 2012, Santos et al. 2015). Hereafter, we refer to this pathway as the Dsr-pathway. Most SRM (with the exception of early diverging archaea) and microorganisms relying on a partial sulfate reduction pathway such as sulfite-, thiosulfate-, and organosulfonate reducers as well as sulfur disproportionating microorganisms utilize in addition DsrD, which is an allosteric activator of DsrAB (Ferreira et al. 2022). Among these enzymes, DsrAB can be used not only as a functional but, with some limitations, also as a phylogenetic marker for SRM. Phylogenetically, this enzyme comprises three major lineages that largely differentiate between (i) reductively-operating DsrAB of archaeal origin, (ii) reductively-operating DsrAB of bacterial origin, and (iii) oxidatively or reverse-operating DsrAB (rDsrAB), which occur in a variety of phototrophic and chemotrophic SOM (Loy et al. 2009, Müller et al. 2015). SOM that rely on rDsrAB for sulfur oxidation also share a number of additional enzymes with SRM, including Sat, AprAB, QmoABC, DsrC, and DsrMKJOP (Dahl 2017, Tanabe and Dahl 2022).

The phylogenetic distinction of reductively and oxidatively operating DsrAB was initially also supported by the presence of additional, presumably SOM-specific enzymes. These include DsrEFH as a sulfur donor protein for DsrC in SOM (Stockdreher et al. 2012) and DsrL as an essential oxidoreductase in sulfur oxidation (Lübbe et al. 2006) that transfers reducing equivalents from rDsrAB to NAD^+^ (Löffler et al. 2020). However, metagenome-assembled genomes (MAGs) from a variety of habitats questioned this clear distinction, with DsrEFH, DsrL, or both being co-encoded together with reductive DsrAB (Anantharaman et al. 2018, Hausmann et al. 2018, Tan et al. 2019, Thiel et al. 2019, Ye et al. 2022). The recent identification of two discrete DsrL types, with DsrL-1 occurring only in SOM, while DsrL-2 occurring in organisms with either a reductive/disproportionating or oxidative sulfur metabolism (Löffler et al. 2020), highlights the difficulties in delineating the energy metabolism solely from genomic data. Functional gene prediction is further complicated by the examples of *Desulfurivibrio alkaliphilus* (Thorup et al. 2017) and the so-called cable bacteria affiliated to the *Desulfobulbaceae* (Pfeffer et al. 2012, Risgaard-Petersen et al. 2015). Both can oxidize sulfide by operating the canonical pathway of sulfate reduction in reverse, including a reductive-type DsrAB, and couple this either with intracellular nitrate reduction in the case of *D. alkaliphilus* (Thorup et al. 2017) or to electrogenic oxygen or nitrate reduction in spatially separated cells along filaments in the case of cable bacteria (Kjeldsen et al. 2019).

Despite these constraints, *dsrAB* gene-based molecular approaches have become an important tool for studying the diversity and ecology of SRM in the environment. First introduced by Wagner et al. 1998, cumulative evidence from a large variety of marine, terrestrial, and man-made environments revealed that the diversity of SRM extends massively beyond cultured representatives in the four bacterial phyla *Desulfobacterota* (formerly known as *Deltaproteobacteria* and *Thermodesulfobacteria*, Waite et al. 2020), *Bacillota* (formerly known as Firmicutes, Oren and Garrity 2021), *Thermodesulfobiota* (Frolov et al. 2023), and *Nitrospirota* (Oren and Garrity 2021) as well as the two archaeal phyla *Thermoproteota* (formerly known as Crenarchaeota, Oren and Garrity 2021) and *Halobacterota* (formerly part of the Euryarchaeota, Rinke et al. 2021). A systematic review of environmental *dsrAB* genes encoding the reductive bacterial-type DsrAB revealed at least 13 lineages at the approximate family level that could not be related to any cultured SRM or higher-rank taxa (Pester et al. 2012, Müller et al. 2015). At the species level, a broad census based on *dsrB* gene amplicon sequencing identified 167 397 species-level operational taxonomic units (OTUs) across 14 different environments (Vigneron et al. 2018). If compared to the approximately 460 described SRM listed in the LPSN database (lpsn.dsmz.de), this means that > 99% of SRM diversity is represented by uncultured microorganisms without taxonomic assignment.

Members of well characterized *Desulfobacterota* (*Desulfobacteraceae, Syntrophobacteraceae, Desulfovibrionaceae, Desulfobulbaceae*) often dominate the SRM community in marine and freshwater surface sediments (Vigneron et al. 2018, Wörner and Pester 2019, Jørgensen 2021) and the uncharted *dsrAB* gene sequence space largely represents low-abundance taxa. However, in certain environments representatives of uncultured *dsrAB* lineages can constitute numerically relevant members of the SRM community (Vigneron et al. 2018), including coastal sediments in the Arctic (Flieder et al. 2021), wetlands (Pester et al. 2012), and deep subsurface marine sediments with active but cryptic sulfur cycling (Leloup et al. 2009), to name a few. Therefore, there is a need to identify these yet unknown SRM and to understand their ecophysiology and evolution. In recent years, an increasing number of new DsrAB-encoding taxa have been discovered by metagenomic surveys of environmental samples and the delineation of MAGs. Here, we provide a systematic review of these novel findings, give insights into the increased diversity of (putative) SRM, and place this in the context of the recently proposed overarching changes to bacterial and archaeal taxonomy (Parks et al. 2018, Parks et al. 2020, Oren and Garrity 2021, Rinke et al. 2021). Detailed overviews of well-studied phyla harboring SRM, including cultured and environmental representatives, have been provided in excellent reviews elsewhere (Rabus et al. 2013, Rabus et al. 2015, Langwig et al. 2022).

## Unprecedented diversity of *Bacteria* and *Archaea* with the potential for dissimilatory sulfate/sulfite metabolism

The number of genomes of uncultivated microorganisms assembled from metagenomes is rapidly growing. In recent years, thousands of MAGs from poorly characterized bacterial and archaeal phyla, including those that still lack cultured representatives (candidate phyla), were recovered from a large variety of environments (Anantharaman et al. 2016, Parks et al. 2017, Parks et al. 2018, Rinke et al. 2021). The vast number of novel MAGs allowed researchers to screen for the genomic potential of a dissimilatory sulfur metabolism in microbial lineages that were previously not linked to such processes. In addition, bioinformatics tools were developed lately to identify genes related to sulfur compound dissimilation, transport and intracellular transfer with confidence and in a high throughput manner (Mendler et al. 2019, Neukirchen and Sousa 2021, Tanabe and Dahl 2022, Zhou et al. 2022). This resulted in a burst of discoveries since 2018. For example, a study by Anantharaman et al. (2018) substantially expanded the known diversity of bacterial and archaeal phyla with the capacity for sulfite/sulfate reduction from 7 to 20 phylum-level lineages. Specifically, members of the *Acidobacteriota, Armatimonadota, Bacteroidota, Verrucomicrobiota*, UBA9089 (*Ca*. Desantisbacteria), SAR324 (*Ca*. Lambdaproteobacteria), *Ca*. Zixibacteriota and *Ca*. Hydrothermarchaeota contained Dsr-pathway genes to perform sulfate/sulfite reduction (Anantharaman et al. 2018). *Chloroflexota* associated with marine sediments (Wasmund et al. 2016) and freshwater subsurface sediments (Hug et al. 2016), newly described members of the *Nitrospirota* recovered from rice paddy soil (Zecchin et al. 2018), enigmatic bacteria of novel candidate phyla such as SZUA-79 (*Ca*. Acidulodesulfobacterales) from a mine drainage with pH ∼2 (Tan et al. 2019), and cryptic candidate phyla like UBA9089 and CG2-30-53–67 (Probst et al. 2017) contribute further to the diversity of bacteria with the potential to reduce sulfate/sulfite. Anaerobic oxidation of methane (AOM) or other alkanes coupled to sulfate reduction was suggested to be performed by microbial consortia of methanotrophic archaea and sulfate-reducing bacteria (Boetius et al. 2000, Knittel and Boetius 2009). The recent finding that some *Halobacterota* (*Archaeoglobaceae*) and *Thermoproteota* (*Ca*. Methanodesulfokores washburnensis) encode both a methanogenesis pathway and the capability to perform sulfate or sulfite reduction, respectively, suggests that sulfur-dependent AOM can be carried out in a single organism, independent from syntrophic interactions (McKay et al. 2019, Wang et al. 2019).

**Figure 2.**
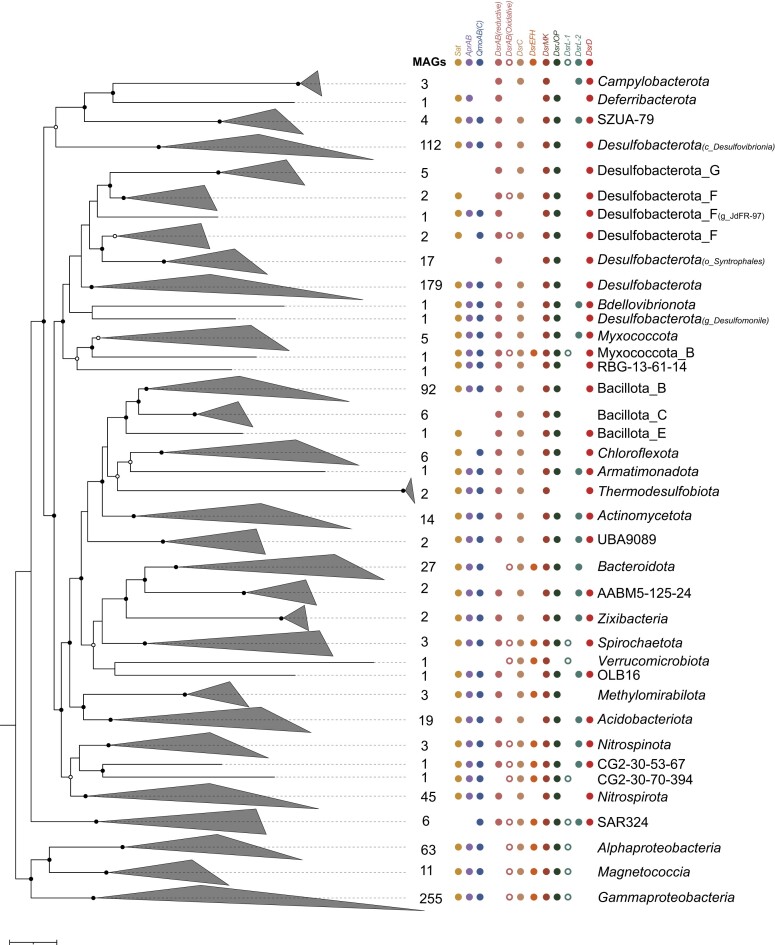
Phylogeny and Dsr-pathway composition of DsrAB-encoding bacteria. Bacterial genome tree inferred from 120 concatenated proteins as based on the GTDB taxonomy (Parks et al. 2018). The phylogenomic tree was inferred from 902 bacterial (metagenome-assembled) genomes. The scale bar indicates 20% sequence divergence. The maximum likelihood tree was constructed with IQ-TREE (Nguyen et al. 2015) using automatic substitution model selection (LG + F + R10) and ultrafast bootstrap analysis (n = 1000). Bootstrap support is indicated by black dots (≥90%) or black circles (70–90%). Within each lineage, the presence of Dsr-pathway encoding genes was indicated if > 30% of *dsrAB*-containing genomes carried the respective genes as inferred by an automated hmm search (Zhou et al. 2022, custom-made pHMM for DsrL: https://github.com/AnantharamanLab/Diao_et_al_2023).

The enormous increase in the phylogenetic breadth of bacteria and archaea with the potential for DsrAB-based dissimilatory sulfate/sulfite reduction is currently missing a systematic overview. Here, we screened all publicly available and functionally pre-annotated genomes and MAGs summarized on the Annotree platform v.1.2.0 (Mendler et al. 2019) for the presence of *dsrAB* genes (http://annotree.uwaterloo.ca, accessed on April 3^rd^, 2023, for bacteria and February 6^th^, 2023, for archaea). This resulted in a total of 902 bacterial and 48 archaeal genomes distributed across 27 and 4 phyla according to the GTDB-Tk taxonomy (Parks et al. 2018, Rinke et al. 2021), respectively (Fig. 2, Fig. 5). Phyla provisionally split by the GTDB release 214 into different sublineages such as *Bacillota* and Bacillota_A to Bacillota_H were counted as one phylum. The retrieved 950 genomes represented 370 isolated species (353 bacterial and 17 archaeal species) and 936 species in total according to the GTDB-Tk taxonomy (Table S1). Since Annotree (Mendler et al. 2019) is based on the Genome Taxonomy Database, which considered only MAGs of > 50% completeness and < 10% contamination (Parks et al. 2018, Rinke et al. 2021), MAGs that did not fulfill these quality criteria were omitted from our analysis. These included, for example, a *Verrucomicrobiota* MAG with early diverging DsrAB (Verrucomicrobia bacterium SbV1) as well as representatives of the Schekmanbacteria (Schekmanbacteria bacterium RBG_13_48_7) and *Chloroflexota* (Chloroflexi bacteria RBG_13_60_13 and RBG_13_52_14) described previously in the literature (Anantharaman et al. 2018).

The identified DsrAB-encoding genomes and MAGs were analyzed in more detail using protein hidden Markov Models (pHMMs) (Zhou et al. 2022, custom-made pHMM for DsrL: https://github.com/AnantharamanLab/Diao_et_al_2023) to search for genes encoding proteins known to be involved in dissimilatory sulfur metabolism (Fig. 1; Tables S1). About half of the identified bacterial genomes were affiliated to the *Desulfobacterota* (35%), *Bacillota* (11%), *Nitrospirota* (5%), and *Thermodesulfobiota* (0.2%), which together represent phyla encompassing all cultured and well-characterized bacterial SRM (Rabus et al. 2013, Rabus et al. 2015). In addition, members of the *Campylobacterota* (i.e. *Desulfurella* spp.) represented cultured thiosulfate-reducing bacteria that employ the Dsr-pathway (Florentino et al. 2017, Florentino et al. 2019). The second largest group of DsrAB-encoding bacterial genomes belonged to the *Pseudomonadota* (36%), including the classes *Alphaproteobacteria, Gammaproteobacteria*, and *Magnetococcia*, and to the *Bacteroidota*, class *Chlorobia* (2%). The two phyla represent cultured and well-characterized SOM with an oxidatively-operating Dsr-pathway (Loy et al. 2009, Dahl 2017). Members of DsrAB-encoding, canonical SRM- or SOM-related lineages also encoded all other Dsr-pathway proteins. The only exceptions to this were DsrL, either of type 1 or 2, and DsrEFH, which were typically encoded by canonical SOM-related lineages but were absent in the overwhelming majority of genomes affiliated to canonical SRM-related lineages, underlining their relevance for sulfur oxidation (Lübbe et al. 2006). Vice versa, DsrD was typically encoded in canonical SRM-related but not in SOM-related lineages, implying its relevance for sulfate/sulfite reduction (Ferreira et al. 2022) (Fig. 2).

The remaining 11% of DsrAB-encoding genomes were spread over 20 different bacterial phyla, with the *Acidobacteriota* (19 MAGs) and *Actinomycetota* (14 MAGs) representing the most prominent groups (Fig. 2). Representatives of the *Acidobacteriota, Zixibacteria, Bdellovibrionota, Armatimonadota*, and the candidate phyla UBA9089 (Desantisbacteria), SZUA-79, OLB16, and AABM5-125-24 were all characterized by the full set of Dsr*-*pathway genes, including *dsrD* as indicator for a reductively operating metabolism and *dsrL* of type 2 (Fig. 2), which is present in organisms with either a reductive/disproportionating or oxidative sulfur metabolism. The same was true for MAGs within the *Actinomycetota* and *Myxococcota*, with three exceptions which are described in more detail below. Representatives of the *Chloroflexota, Deferribacterota*, and candidate phylum RBG-13–61-14 also encoded DsrD but lacked *dsrL* genes. Since most of the other Dsr*-*pathway encoding genes could be recovered for the latter three phyla, a reductively operating pathway is indicated here as well. Members of the *Methylomirabilota* (previously assigned to *Candidatus* Rokubacteria, Anantharaman et al. 2018) lacked both *dsrD* and *dsrL* genes but belong to the group of microorganisms with the earliest diverging DsrAB (Fig. 2). The group of bacteria and archaea with early diverging DsrAB consistently lacks *dsrD* and *dsrL* genes but contains cultured representatives with a reductively operating Dsr*-*pathway (Anantharaman et al. 2018, Ferreira et al. 2022). In summary, members of fourteen phyla without cultured SRM (representing 7% of all recovered bacterial genomes) encode the full enzyme complement to perform dissimilatory sulfate/sulfite reduction. In contrast, representatives of the *Verrucomicrobiota* and candidate phylum CG2-30-70–394 lacked the *dsrD* gene but encoded DsrL-1, which resembles the situation in canonical SOM and is indicative of an oxidatively operating sulfur metabolism.

**Figure 3.**
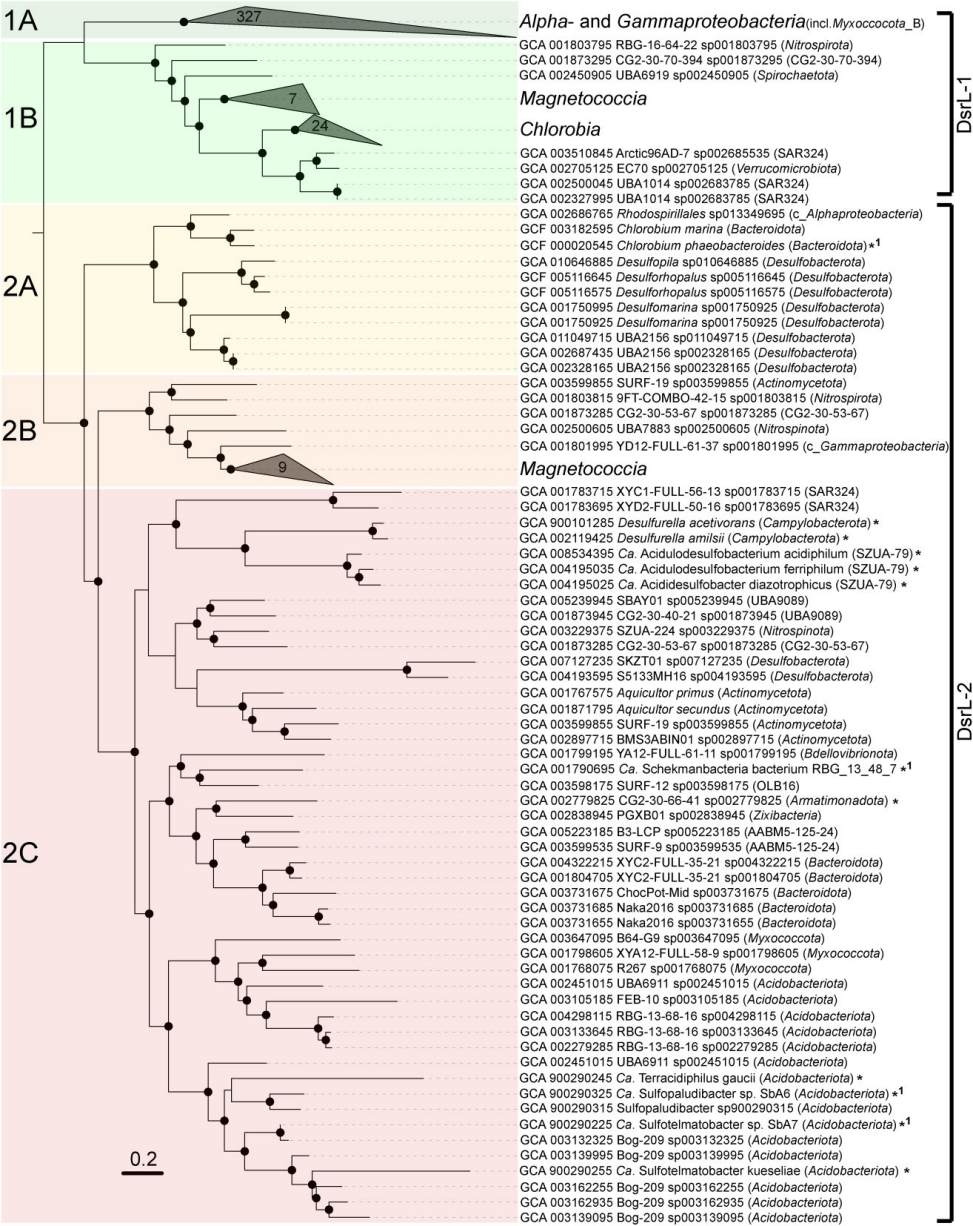
Phylogenetic analysis of bacterial DsrL proteins. The maximum likelihood tree was inferred from 438 DsrL proteins and constructed with IQ-TREE (Nguyen et al. 2015) using automatic substitution model selection (Q.pfam + I + R9) and ultrafast bootstrap analysis (n = 1000). Bootstrap support is shown by black dots (≥90%). DsrL sequences marked with an asterisk were not detected by the custom-made pHMM for DsrL (https://github.com/AnantharamanLab/Diao_et_al_2023), but have been previously described in the literature (Hausmann et al. 2018, Löffler et al. 2020). Additional DsrL sequences (*1) were collected from MAGs with low completeness, which were not included in our MAG analysis.

The situation was more complex in members of the *Nitrospirota, Nitrospinota, Spirochaetota, Bacteroidota*, and SAR324. In these five phyla, different MAGs of the same phylum carried different gene combinations of the Dsr*-*pathway. Within the *Nitrospirota*, the majority of MAGs encoded DsrD but lacked genes encoding DsrL, including cultured SRM of the genus *Thermodesulfovibrio* (Zecchin et al. 2018). However, there were two MAGs that lacked the *dsrD* gene but encoded either DsrL-1 (f_RBG-16–64-22) or DsrL-2 (f_9FT-COMBO-42–15) indicating an oxidatively operating Dsr-pathway (Fig. 3, Supplementary Table S1). The opposite was true for the *Bacteroidota*. Here, most MAGs and genomes of cultured representatives belonged to the class *Chlorobia* (family *Chlorobiaceae*), which represent canonical SOM and carried gene combinations of the Dsr-pathway typical for SOM (no *dsrD, dsrL-1* or *dsrL-*2). However, five representatives of the *Bacteroidota* family UBA2268 (class *Kapabacteria*), encoded both DsrD and DsrL-2 (which was clearly distinct from DsrL of the *Chlorobiaceae*) indicating a reductively operating Dsr-pathway. *In situ* transcriptional profiles of UBA2268 MAGs recovered from hot spring microbial mats clearly supported a reductively operating Dsr-pathway activated under anoxic conditions (for details see below, Thiel et al. 2019). For members of the *Nitrospinota* and *Spirochaetota*, MAGs were more evenly distributed and either carried gene combinations indicative of a reductive (*dsrD* along with *dsrL*2 or no *dsrL*) or oxidative sulfur metabolism (no *dsrD* but *dsrL*-1 or *dsrL*-2). Furthermore, our analysis recovered six SAR324 members with different gene combinations of the Dsr-pathway. Two SAR324 MAGs, which affiliated to the provisional family XYD2_FULL-50–16, were recovered from the terrestrial subsurface with an indicated reductive sulfur metabolism (*dsrD* along with *dsrL* 2). The remaining four MAGs (f_NAC60-12) were recovered from marine environments with an indicated oxidative sulfur metabolism (no *dsrD, dsrL*-1). The latter coincides well with reports of Dsr-pathway encoding SAR324 from oxygenated deep ocean waters (Swan et al. 2011), hydrothermal vent plumes (Sheik et al. 2014), and marine oxygen minimum zones (van Vliet et al. 2021).

**Figure 4.**
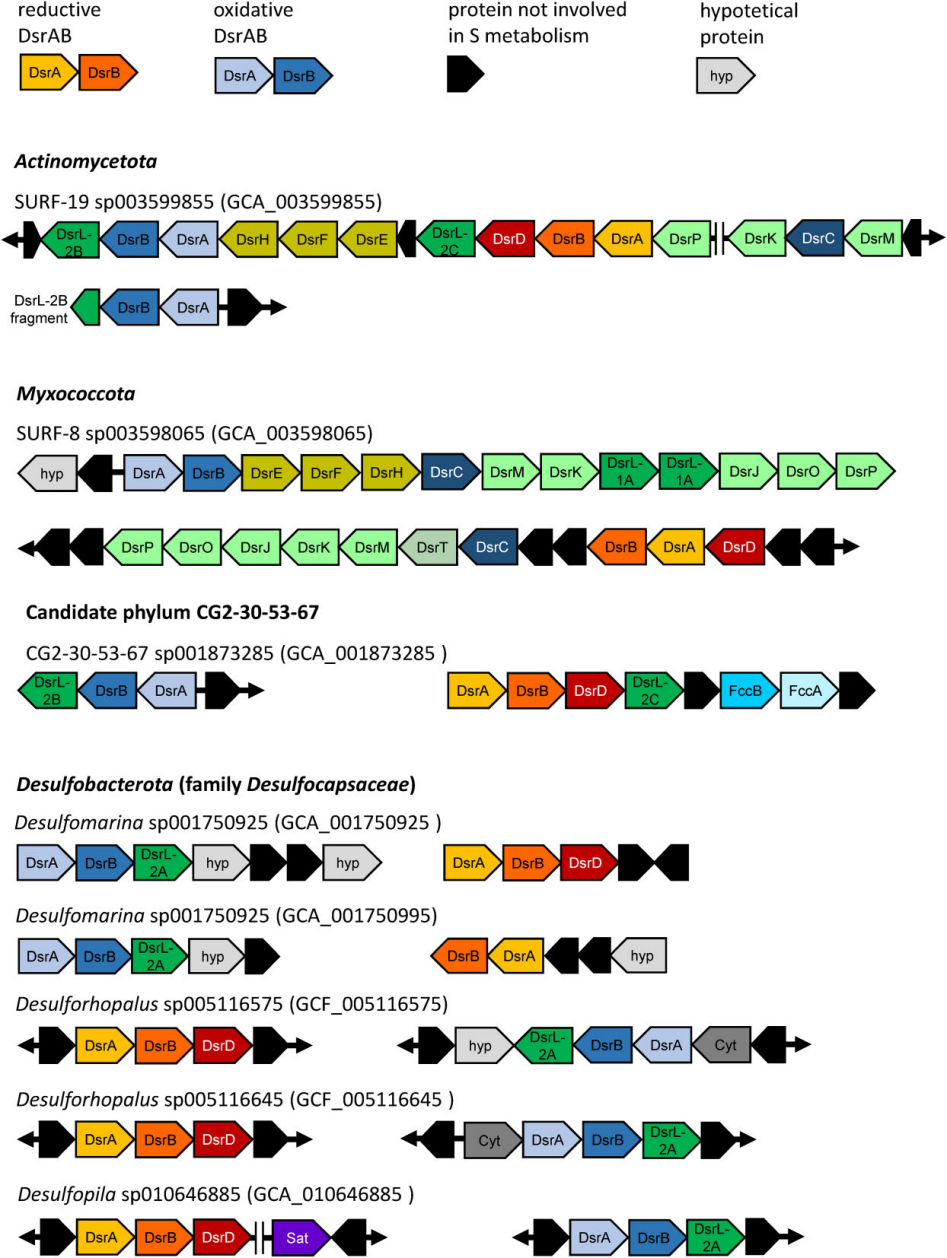
Organization of *dsr* gene clusters in MAGs encoding both reductive and oxidative bacterial-type DsrAB.

An unusual gene combination was observed for eight MAGs spread over the phyla *Actinomycetota, Myxococcota*, CG2-30-53–67 (one MAG each) and the *Desulfobacterota* (five MAGs within the family *Desulfocapsaceae*). Here, at least two different bacterial-type DsrAB, including always one reductive and one oxidative one, were encoded on the same genome (Fig. 4 , Table S1). The most striking example was *Actinomycetota* MAG GCA_003 599 855, which was recovered from a 1.5 km deep terrestrial aquifer (Momper et al. 2017) and encoded two different oxidative DsrAB and one reductive DsrAB. On a single contig, both reductive and oxidative DsrAB were encoded in close proximity. The reductive DsrAB was encoded upstream of DsrD and DsrL-2, with the latter falling into the major DsrL-2 cluster. The oxidative DsrAB was encoded just four genes further downstream and flanked by genes encoding DsrEFH (upstream) and a second copy of DsrL-2 (downstream). The latter clustered together with DsrL-2 of *Magnetococcia, Nitrospinota* family UBA7883 and *Nitrospirota* family 9FT-COMBO-42–15, which all have a verified or indicated oxidative sulfur metabolism (Löffler et al. 2020). On a separate contig, the second oxidative DsrAB was encoded just upstream of a fragmented *dsrL*-2 gene located at the end of the contig (Fig. 4). From the same terrestrial subsurface habitat, Myxococcota_B MAG GCA_003 598 065 was recovered as another interesting example (Momper et al. 2017). It encoded reductive DsrAB downstream of *dsrD* and upstream of *dsrCTMKJOP* and on a separate contig oxidative DsrAB just upstream of *dsrEFHCMKLLJOP*. Interestingly, DsrL was of type 1 and encoded by two gene copies in direct vicinity. An additional *Myxococcota* MAG (GCA_003 153 055) encoded oxidative DsrAB as well but was missing further genes indicative of a reductive or oxidative metabolism. Yet another unusual representative of the terrestrial deep subsurface was the single MAG representing candidate phylum CG2-30-53–67 (Table S1), which was recovered from deep groundwater (Probst et al. 2017). Also here, the reductive DsrAB was encoded upstream of DsrD and DsrL*-*2, with the latter falling into the major DsrL-2 cluster. The oxidative DsrAB was encoded upstream of a second copy of DsrL*-*2, which clustered together with DsrL-2 of *Magnetococcia* (Figs 3 and 4). As suggested before (Löffler et al. 2020), this bacterium is likely capable of switching the direction of dissimilatory sulfur metabolism by regulating the different types of DsrABL. The same is likely true for the *Actinomycetota* MAG described above.

**Figure 5.**
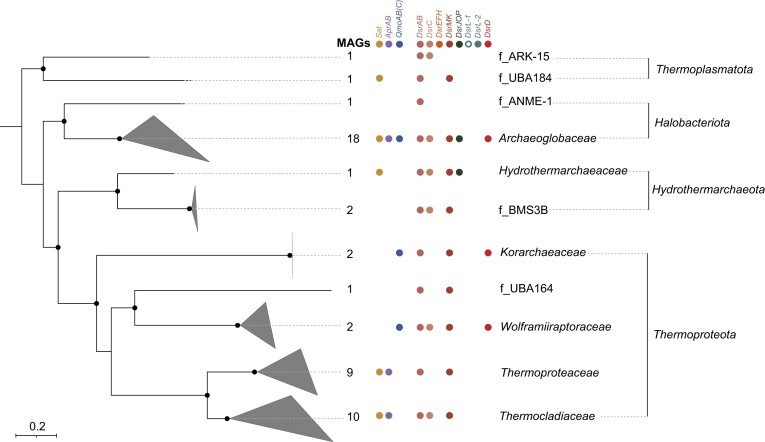
Phylogeny and Dsr-pathway composition of DsrAB-encoding archaea. Archaeal genome tree inferred from 122 concatenated proteins as based on the GTDB taxonomy (Rinke et al. 2021). The phylogenetic tree was inferred from 48 archaeal (metagenome assembled) genomes. The scale bar indicates 20% sequence divergence. The maximum likelihood tree was constructed with IQ-TREE (Nguyen et al. 2015) using automatic substitution model selection (LG + F + R6) and ultrafast bootstrap analysis (n = 1000). Bootstrap support is indiacted by black dots (≥90%) or black circles (70%–90%). Within each lineage, the presence of Dsr-pathway encoding genes was indicated if > 30% of *dsrAB*-containing genomes carried the respective genes as inferred by an automated pHMM search (Zhou et al. 2022).

The five unusual MAGs from the family *Desulfocapsaceae* within the *Desulfobacterota* were spread over the three genera *Desulforhopalus, Desulfomarina*, and *Desulfopila* (Fig. 4, Table S1). A uniting feature of these MAGs was their recovery from oxic-anoxic transition zones in marine surface sediments, either at methane seeps or tidal sediments. A second uniting feature was the genomic organization of the different *dsrAB* gene sets. Reductive DsrAB was always encoded upstream of DsrD and oxidative DsrAB always upstream of DsrL-2 (Fig. 4), which clustered together with DsrL-2 of canonical SOM of the genus *Chlorobium* (Fig. 3). Both *dsrAB* gene sets were always recovered on separate contigs and did not form operons with other Dsr-pathway genes such as *dsrMKJOP, aprAB, qmoABC*, and *sat*. Here, a switch of the direction of dissimilatory sulfur metabolism might be regulated by differentially forming DsrABD or DsrABL-2. Interestingly, three additional *Desulfobacterota* MAGs affiliated to the provisional genus UBA2156 encoded oxidative DsrAB only again along with DsrL-2, which clustered with DsrL-2 of canonical SOM of the genus *Chlorobium* (Fig. 3). Since one of these MAGs also encoded DsrD, it remains unclear whether the genes encoding reductive DsrAB were missed by the assembly and binning process. Vice versa, a misassembly or false binning cannot be excluded for any of the above-mentioned MAGs carrying unusual *dsrAB* gene combinations. However, all these MAGs were of high quality with estimated contaminations ranging from 0.7% to 6.4% (3.0 ± 2.0%, average ± standard deviation) and estimated completeness ranging from 77% to 99% (92 ± 8%, average ± standard deviation). The combination of this high binning quality with the recovery of such unusual *dsrAB* gene combinations in multiple studies from various environments and several phylogenetic lineages makes the likelihood quite high that these MAGs represent real microorganisms awaiting discovery using cultivation approaches.

Based on the findings described above, we propose to further subdivide the DsrL-2 cluster into three phylogenetically distinct subclusters to guide genome annotations. Subclusters DsrL-2A and DsrL-2B encompass (i) canonical SOM of the *Bacteroidota* and *Pseudomonadota*, (ii) *Nitrospinota* and *Nitrospirota* MAGs with an indicated oxidative sulfur metabolism, (iii) and MAGs encoding oxidative and reductive DsrAB on the same genome. For the latter group, DsrL-2A and DsrL-2B were always encoded just downstream of oxidative DsrAB, with the respective genes being part of the same operon (Fig. 4). Therefore, we propose DsrL-2A and DsrL-2B to function as an indicator for an oxidative sulfur metabolism if encoded in close proximity to oxidative DsrAB. In contrast, subcluster DsrL-2C encompasses all MAGs with an indicated reductive sulfur metabolism as evidenced by encoding DsrD and reductive DsrAB but not oxidative DsrAB. Here, we propose DsrL-2C to function as an indicator for a reductive sulfur metabolism if encoded in close proximity to reductive DsrAB. DsrL-1 subclusters A and B were already defined by Löffler et al. 2020, and encompass canonical SOM of the *Alpha*- and *Gammaproteobacteria* for subcluster DsrL-1A and canonical SOM of the *Chlorobia* and *Magnetococcia* as well as MAGs with an indicated oxidative sulfur metabolism for subcluster DsrL-1B. Along these lines, we examined the occurrence of DsrEFH among all recovered MAGs. In the overwhelming majority, DsrEFH was encoded in SOM and MAGs with an indicated oxidative sulfur metabolism and absent in SRM and MAGs with an indicated reductive sulfur metabolism (Fig. 2). Also, in MAGs that encoded both reductive and oxidative DsrAB, DsrEFH was always encoded along with oxidative DsrAB (Fig. 4). However, there were two lineages that represented exceptions to this general pattern. Two *Actinomycetota* within the genus *Aquicultor* and two SAR324 affiliated to the provisional family XYD2_FULL-50–16 encoded both reductive DsrAB and DsrEFH on the same genome (Table S1), making DsrEFH an imperfect predictor for an indicated reductive or oxidative sulfur metabolism.

Archaea encoding the Dsr*-*pathway are currently characterized by a solely reductively operating Dsr-pathway. Among the 48 archaeal genomes studied, 67% belonged to cultured representatives with a known sulfate-, sulfite-, or thiosulfate-reducing metabolism within the phyla *Thermoproteota* (members of the genera *Caldivirga, Thermoproteus, Thermocladium, Vulcanisaeta*, and *Pyrobaculum*) and *Halobacteriota* (*Archaeoglobus* spp.). The remaining MAGs expanded the diversity of archaea encoding a Dsr*-*pathway to include two additional phyla (*Hydrothermarchaeota* and *Thermoplasmatota*) and four additional families in the *Thermoproteota* (three families) and *Halobacterota* (one family). Most of the DsrAB-encoding archaea either represented cultured thermophiles or their MAGs were retrieved from high-temperature environments (e.g. hot springs, hydrothermal vent fluids) including their deposits (e.g. deep sea hydrothermal vent field site). The only exceptions were two *Halobacteriota* MAGs (GCA_002507545, GCA_002494625) and one *Thermoplasmatota* MAG (GCA_002503985) assembled from marine sediment or soil metagenomes, respectively (Parks et al. 2017). Notably, at least two of the families encoding the Dsr-pathway (*Korarchaeaceae*, phylum *Thermoproteota; Archaeoglobaceae*, phylum *Halobacteriota*) were represented by MAGs (McKay et al. 2019, Wang et al. 2019) that additionally encode the complete pathway for (reverse) methanogenesis. Based on these findings, it was postulated that anaerobic methane oxidation coupled to sulfate/sulfite reduction might also operate in single microorganisms (McKay et al. 2019, Wang et al. 2019) as opposed to the standard model of syntrophic associations (Knittel and Boetius 2009).

## Genome-centric metagenomics anchors and expands *dsrAB*-based functional and taxonomic assignment

**Figure 6.**
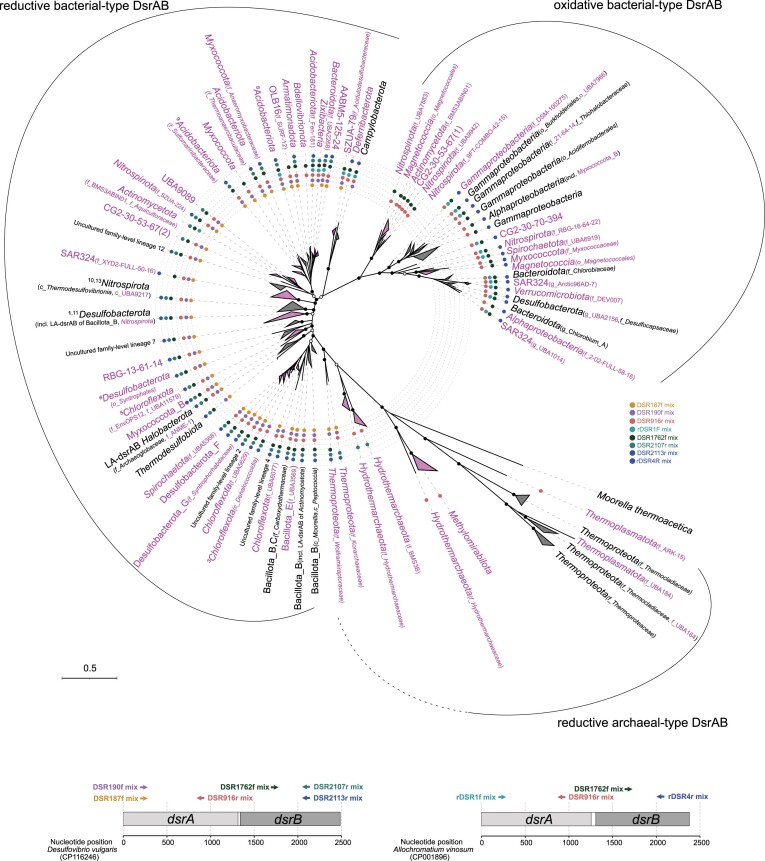
Maximum likelihood phylogeny of DsrAB sequences derived from (metagenome assembled) genomes and environmental surveys. Clades represented by a majority of DsrAB-encoding (metagenome assembled) genomes not affiliated to canonical SRM or SOM are shown in magenta. The coverage of inferred phylogenetic clades by published broad-range PCR primers (≥75% of sequences in a clade; 1 mismatch allowed) is indicated by colored dots. The binding positions of the evaluated primers is indicated at the bottom using *dsrAB* of *Desulfovibrio vulgaris* or *Allochromatiom vinosum* as model organism of *dsrAB* primers designed to target bacterial-type *dsrAB* encoding the reductive or oxidative enzyme version, respectively. The maximum likelihood tree was constructed using deduced DsrAB amino acid sequences with IQ-TREE (Nguyen et al. 2015) using automatic substitution model selection (LG + R10) and ultrafast bootstrap analysis (n = 1000). Bootstrap support is indicated by black dots (≥90%) or black circles (70–90%). The tree was inferred from 613 representative DsrAB sequences with an indel filter covering 571 amino-acid positions: 346 representative DsrAB sequences were taken from a curated DsrAB database including 7921 pure culture and environmental sequences (as based on Müller et al. 2015) and amended with 267 DsrAB sequences derived from (metagenome assembled) genomes representing novel phylogenetic clades. Scale bar indicates 50% sequence divergence. Clades containing taxonomically resolved uncultured family-level DsrAB lineages are indicated by a superscript number based on the following denomination: 1, uncultured family-lineage 1; 3, uncultured family-lineage 3; 5, uncultured family-lineage 5; 6, uncultured family-lineage 6; 8, uncultured family-lineage 8; 9, uncultured family-lineage 9; 10, uncultured family-lineage 10; 11, uncultured family-lineage 11; 13 uncultured family-lineage 13. Please note that the numbers in brackets behind candidate phylum CG2-30-53–67 represent the two diverging *dsrAB* copies carried by the single MAG representing this phylum. LA-*dsrAB*, laterally acquired *dsrAB*.

Approaches based on *dsrAB* gene sequence analysis have been extensively used to study the ecology of SRM and in part also SOM. These surveys were based on the assumption that there is a clear phylogenetic separation between DsrAB present in archaea with a reductive sulfur metabolism, bacteria with a reductive sulfur metabolism, and bacteria with an oxidative sulfur metabolism. We used the expanded diversity of Dsr-pathway encoding microorganisms described above along with the indicated reductively or oxidatively operating direction of their sulfur metabolism to explore their DsrAB phylogeny. Our analysis showed that the distinction of archaeal reductively-operating DsrAB, bacterial reductively operating DsrAB, and bacterial oxidatively-operating DsrAB still largely holds true. While archaeal reductively operating DsrAB and bacterial oxidatively-operating DsrAB formed monophyletic clusters in our analysis (with the exception of laterally acquired *dsrAB* genes, see below), bacterial reductively-operating DsrAB were spread between these two clusters in a bush-like manner (Fig. 6). This is consistent with a recent phylogenetic analysis of DsrAB including already parts of the novel *dsrAB* gene sequence space discovered in MAGs from various habitats (Anantharaman et al. 2018). However, it does not reproduce anymore the monophyletic separation of reductively-operating DsrAB observed in phylogenetic analyses based mainly on canonical SRM and PCR-derived *dsrAB* gene sequences of environmental studies (e.g. Müller et al. 2015). Nevertheless, the distinction of reductive bacterial-type DsrAB in our (metagenome assembled) genome census was not only anchored by canonical sulfate/sulfite-reducing bacteria but contained in addition exclusively phyla whose representative MAGs encoded DsrD (in combination with or without DsrL-2C) strongly indicating a reductive-type sulfur metabolism as well. The only exception were the few MAGs encoding both reductive and oxidative bacterial-type DsrAB. However, also in these MAGs DsrD was always encoded downstream of reductive bacterial-type DsrAB (Fig. 4). Furthermore, the demarcation to oxidative bacterial-type DsrAB was well supported by the latest diverging reductive DsrAB cluster that contained DsrAB of *Desulfurella amilsii* (*Campylobacterota*) as an organism capable of growth by thiosulfate reduction (Fig. 6).

Previous PCR-based *dsrAB* gene studies described thirteen lineages of reductive bacterial-type DsrAB that represented approximate family level groups of uncultured microorganisms that could not be assigned to known taxa (Pester et al. 2012, Müller et al. 2015). Members of some of these groups were identified as abundant and active in different marine and freshwater habitats (e.g. Pester et al. 2012, Müller et al. 2015, Pelikan et al. 2016, Wörner and Pester 2019). Based on previous findings and the phylogenomic survey of this study, we summarize the currently known taxonomic classification of these uncultured family-level DsrAB lineages (Fig. 6, Table 1). DsrAB sequences of uncultured lineages 3 and 5 were found in members of the *Chloroflexota*. Lineage 3 members were represented by a single-cell amplified genome (SAG) recovered from deep marine subsurface sediments (Wasmund et al. 2016). Because of the low coverage of this SAG (46%, Wasmund et al. 2016), it was not part of our genome collection but was considered in our DsrAB analysis (Fig. 6, class *Dehalococcoidia*). *Chloroflexota* representing uncultured family lineage 5 were recovered from hydrothermal sediments and a bioreactor (MAG collection of Parks et al. 2017 and Zhou et al. 2020). Uncultured family lineage 6 belongs to the *Desulfobacterota* (order *Syntrophales*) and DsrAB sequences of uncultured lineages 8 and 9 were found in MAGs of terrestrial and marine *Acidobacterota*, respectively (Hausmann et al. 2018, Flieder et al. 2021). Sequences of DsrAB lineages 10 and 13 have been uncovered in *Nitrospirota* genomes from an aquifer system (Anantharaman et al. 2016). Furthermore, uncultured family lineages 1 and 11 cluster within the *Desulfobacterota*. While uncultured family lineages 1 still has an unresolved family affiliation, uncultured family lineage 11 grouped next to laterally acquired *dsrAB* genes of *Nitrospirota* affiliated to provisional family SM23-35. In summary, the affiliation of 8 of the 13 uncultured family-level DsrAB lineages could be resolved using (meta-)genome targeted approaches. However, the affiliation of uncultured family lineages 2, 4, 7, and 12 still awaits its discovery.

**Table 1.** Overview of uncultured family-level DsrAB lineages as proposed by Müller et al. (2015) and their corresponding GDTB-Tk taxonomy.

Uncultured family-level lineages	GTDB-Tk taxonomy	Reference
Uncultured family-level lineage 1	p_*Desulfobacterota*	This study
Uncultured family-level lineage 2	Not resolved	\
Uncultured family-level lineage 3	p_*Chloroflexota*; c_*Dehalococcoidia*	Wasmund et al. 2016
Uncultured family-level lineage 4	Not resolved	\
Uncultured family-level lineage 5	p_*Chloroflexota*; c_*Anaerolineae*; o_*Anaerolineales*	This study
Uncultured family-level lineage 6	p_*Desulfobacterota*; c_*Syntrophia*; o_*Syntrophales*	This study
Uncultured family-level lineage 7	Not resolved	\
Uncultured family-level lineage 8	p_*Acidobacteriota*	Hausmann et al. 2018
Uncultured family-level lineage 9	p_*Acidobacteriota*; c_*Thermoanaerobaculia*; o_*Thermoanaerobaculales*; f_FEB-10	Flieder et al. 2021
Uncultured family-level lineage 10	p_*Nitrospirota*; c_UBA9217; o_UBA9217; f_UBA9217	Anantharaman et al. 2016
Uncultured family-level lineage 11	p_*Desulfobacterota*	This study
Uncultured family-level lineage 12	Not resolved	\
Uncultured family-level lineage 13	p_*Nitrospirota*; c_*Thermodesulfovibrionia*; o_UBA6902; f_UBA6902	Anantharaman et al. 2016

**Figure 7.**
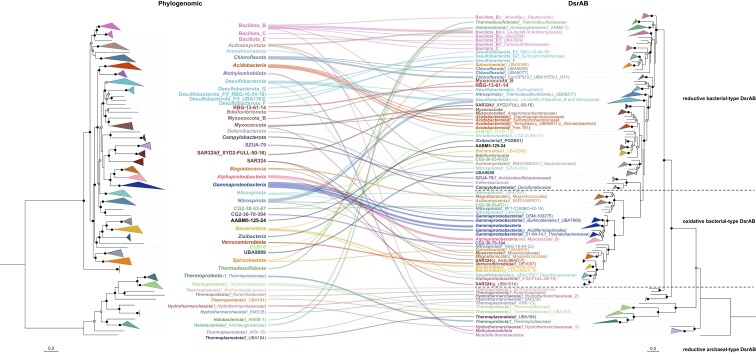
Comparison of phylogenomic and DsrAB trees for microorganisms representing all inferred DsrAB-encoding archaeal and bacterial lineages. The phylogenomic tree was inferred from a set of 43 conserved single-copy marker genes obtained with CheckM (Parks et al. 2015) using 38 representative archaeal and 207 representative bacterial (metagenome-assembled) genomes. The phylogenomic maximum likelihood tree was constructed with IQ-TREE (Nguyen et al. 2015) using automatic substitution model selection (LG + R10) and ultrafast bootstrap analysis (n = 1000). The DsrAB tree was constructed using 269 deduced DsrAB amino acid sequences, which were extracted from 245 representative (metagenome-assembled) genomes. The DsrAB maximum likelihood tree was constructed with IQ-TREE (Nguyen et al. 2015) using automatic substitution model selection (LG + R8) and ultrafast bootstrap analysis (n = 1000). Bootstrap support is indicated by black dots (≥90%) or black circles (70%–90%). For both trees, the scale bars indicates 20% sequence divergence.

Several studies have provided evidence that the distribution of *dsrAB* genes among extant microorganisms is represented by a combination of divergence through speciation, functional diversification and lateral gene transfer (LGT) (Klein et al. 2001, Zverlov et al. 2005, Loy et al. 2008, Müller et al. 2015, Anantharaman et al. 2018). Well documented examples are the laterally acquired *dsrAB* genes of a group of *Desulfotomaculum* spp. (*Bacillota*) from *Desulfobacterota* (Klein et al. 2001, Zverlov et al. 2005) or the bacterial origin of reductive DsrAB in members of the archaeal genus *Archaeoglobus* (*Halobacteriota*) (Müller et al. 2015). Based on our extended analysis, we conclude that 14 major taxa of Dsr-pathway encoding microorganisms likely acquired *dsrAB* genes in multiple lateral gene transfer events. These encompass besides the *Bacillota* and *Halobacterota* also the *Methylomirabilota, Chloroflexota, Alphaproteobacteria, Magnetococcia, Hydrothermarchaeota, Desulfobacterota, Actinomycetota, Nitrospirota, Nitrospinota, Bacteroidota, Spirochaetota, Myxococcota*, and candidate phyla CG2-30-53–67 and SAR324 (Fig. 7). The latter seven are especially interesting because they harbor members with either reductive DsrAB, oxidative DsrAB, or both (Fig. 6, Fig. 7). Analysis of our extended *dsrAB* gene dataset could not reproduce the postulated LGT of bacterial *dsrAB* to archaea of the families *Wolframiiraptoraceae* (previously referred to as Aigarchaeota pSL4) and *Korarchaeaceae* (*Ca*. Methanodesulfokores washburnensis) (Müller et al. 2015, McKay et al. 2019) despite them showing higher similarity to reductive bacterial-type DsrAB of *Bacillota* than to reductive DsrAB of all other archaea (Fig. 6). More in-depth phylogenetic studies will have to show whether lateral gene transfer of *dsrAB* occurred in *Wolframiiraptoraceae* and the *Korarchaeaceae* as well.

With the updated *dsrAB* gene database provided in this review, *dsrAB*-based marker gene surveys will greatly benefit as sequences can be better taxonomically anchored. To this end, we provide an updated *dsrA* and *dsrB* gene reference database including the sequences from 902 bacterial and 48 archaeal genomes and MAGs analyzed in this study (available under https://www.arb-silva.de/projects/dsrabsilva/), which will be useful for *dsrAB* gene amplicon sequencing analyses (e.g. Müller et al. 2015, Pelikan et al. 2016, Vigneron et al. 2018, Wörner and Pester 2019). We further evaluated the coverage of those genomes and MAGs with primer sets designed to target the *dsrA* and *dsrB* gene encoding either reductive bacterial-type (Pelikan et al. 2016) or oxidative bacterial-type (Loy et al. 2009, Müller et al. 2015) DsrAB (Tables S2). A good coverage of near full-length bacterial-type *dsrAB* encoding the reductive version of the enzyme can be achieved using the primers DSR190f mix (88%) and DSR2107r mix (88%). This covers the great majority of (putative) SRM (Fig. 6; see Table S3 for details). For amplicon sequencing, we can confirm the recommendation of Pelikan et al. (2016) to use the primer pair DSR1762f mix and DSR2107r mix. They cover most (97% and 88%, respectively) of the bacterial-type *dsrB* genes that encode the reductive enzyme version (Fig. 6; Table S3). However, for extended coverage of the few MAGs within the *Bdellovibrionota, Campylobacterota, Deferribacterota*, SAR324, SZUA-79, OLB16, or AABM5-125-24 new primer variants will need to be designed for both near-full length and short *dsr(A)B* amplicons (Table S3). Primer pairs rDSR1f mix and rDSR4r mix were designed to amplify near full-length bacterial-type *dsrAB* encoding the oxidative enzyme version (Loy et al. 2009). They have an acceptable coverage (68% and 95%, respectively), but do not cover a considerable fraction of the oxidative DsrAB-encoding *Alphaproteobacteria, Gammaproteobacteria*, and *Magnetococcia* as well as oxidative DsrAB-encoding members of the *Nitrospinota, Nitrospirota*, and candidate phylum CG2-30-53–67. However, the majority of bacterial-type *dsrB* genes encoding the oxidative enzyme version is covered by the primer pairs DSR1762f mix (94%) and rDSR4r mix (95%), which can be used for short-read amplicon sequencing (Table S3). No primers were published so far to specifically amplify *dsrAB* of archaeal origin.

## Insights into the ecophysiology of newly discovered SRM

For most of the newly discovered microorganisms possessing a Dsr-pathway only a (partial) genome sequence is available so far. Even though their genomic context provides clues about a reductively or oxidatively operating dissimilatory sulfur metabolism, we still miss a large part of their actual physiology. Enriching and isolating these microorganisms into culture will remain the best approach to understand their biology but will also take time. An alternative to cultivation is to understand the ecophysiology of these newly discovered SRM (and SOM) in their natural setting using controlled experimental setups along with studying their activity responses at the transcriptome and/or proteome level or using isotope labeling techniques at the population or single-cell level. In the following, we describe several examples, where this has been achieved.

*Acidobacteriota* encoding a Dsr-pathway were first discovered in pristine low-sulfate environments including peatlands (Hausmann et al. 2018) and aquifers (Anantharaman et al. 2018) but also in acidic sulfide mine waste rock sites (Anantharaman et al. 2018) and later in marine surface sediments (Coskun et al. 2019, Flieder et al. 2021). Clone library-based studies targeting *dsrAB* genes extend the habitat range to deep marine sediments below the sulfate-methane transition zone and high-temperature environments (reviewed in Pester et al. 2012, Müller et al. 2015). A few studies succeeded to investigate the encoded metabolic potential, the transcriptional activity, as well as the abundance and distribution of Dsr-pathway encoding *Acidobacteriota* in more detail.

Peatland *Acidobacteriota* encoding a Dsr-pathway were studied in detail in a small acidic fen in the Fichtel mountains located in Central Europe. Here, they make up roughly two thirds of microorganisms encoding reductive bacterial-type DsrAB (Pelikan et al. 2016) and contribute a considerable fraction to the overall microbial peat soil transcriptome (>2% of all mRNA reads; Hausmann et al. 2018), implying a predominant role in the cryptic sulfur cycle of this habitat. They are affiliated to four different families (*Acidobacteriaceae*, SBA1, *Bryobacteraceae*, and UBA7540) within the class *Terriglobia* (comprising former uncultured *dsrAB* family-level lineage 8) with some recovered MAGs encoding the full canonical pathway of sulfate reduction while others harboring only genes for sulfite reduction. The latter encoded in addition enzymes that can liberate sulfite from organosulfonates, implying organic sulfur compounds as complementary energy sources. Interestingly, these *Acidobacteriota* encoded also the full respiratory chain for aerobic respiration including low and high affinity terminal oxidases as well as a large enzymatic repertoire for polysaccharide degradation and sugar utilization. In addition, capabilities for a fermentative lifestyle and hydrogen oxidation were encoded as well. This “Swiss army knife”-array of potential energy metabolism variants opens up a lot of possibilities how these Dsr-pathway encoding *Acidobacteriota* may cope with the fluctuating redox conditions in peat soils. The redox state of these typically water-saturated soils can change dramatically and is mainly driven by changes in the water table through rainfall and droughts. In addition, lateral flow of water can heavily influence the topography of redox gradients in peat soils through space and time (Frei et al. 2012, Pester et al. 2012). In response, Dsr-pathway encoding *Acidobacteriota* were postulated to be capable of switching from a sulfate-reducing or, in case of sulfate shortage, fermentative lifestyle under anoxic conditions to aerobic respiration under oxic conditions using polysaccharides or low-molecular weight organic compounds as substrates (Hausmann et al. 2018). Especially the potential use of polysaccharides under sulfate reducing conditions would differentiate them from canonical SRM, which are not able to degrade organic polymers (Rabus et al. 2013). Also, a reversal of the Dsr-pathway for sulfur oxidation in combination with aerobic respiration was proposed (Hausmann et al. 2018), although the genomic context suggests rather a reductively operating sulfur metabolism (see above).

Peat soil incubations under controlled substrate and sulfate supply may provide insights into the postulated metabolism of DsrAB-encoding *Acidobacteriota*. When peat soil was incubated anoxically with individual fermentation intermediates (formate, acetate, propionate, lactate or butyrate) with and without externally supplied sulfate, Dsr-pathway encoding *Acidobacteriota* showed a steady transcriptional activity including all genes of the Dsr*-*pathway. However, there was no significant increase of transcriptional activity triggered by either one of the individually supplied low-molecular weight compounds indicating that the activity of the respective *Acidobacteriota* rather relied on organic substances already present in the peat itself (Hausmann et al. 2018). Extending upon these initial results, Dyksma and Pester (2023) incubated peat soil in a bioreactor setting under alternating oxic (50% air saturation) and anoxic conditions and a steady supply of pectin as an abundant terrestrial plant polysaccharide. Indeed, a Dsr-pathway encoding Acidobacterium differentially expressed the full canonical pathway of sulfate reduction under anoxic conditions and the full respiratory chain under oxic conditions providing experimental evidence that facultatively anaerobic SRM within the *Acidobacteriota* exist (Dyksma and Pester, 2023). Similar results were already indicated in studies on model SRM within the *Desulfobacterota*, i.e. *Desulfovibrio* species, albeit at much lower oxygen concentrations. *Desulfovibrio* spp. typically encode high-affinity *bd*-type terminal oxidases only, which are implied to function in oxygen detoxification rather than aerobic growth (Santana 2008, Ramel et al. 2015). When grown in semi-solid media within an oxygen gradient, *Desulfovibrio magneticus* formed a visible band at the oxic-anoxic interface in the absence of sulfate and the authors interpreted this as micro-oxic growth coupled to oxygen respiration (Lefèvre et al. 2016). Aerotactic band formation was also observed for *Desulfovibrio desulfuricans* in oxygen gradients within a diffusion chamber (Fischer and Cypionka, 2006) and active oxygen reduction as a defense strategy to re-establish anoxic conditions have been reported for *Desulfovibrio, Desulfomicrobium* and *Desulfobulbus* spp. (Brune et al. 2000, Cypionka 2000, Sass et al. 2002, Mogensen et al. 2005). In a more detailed study, a strain of *Desulfovibrio vulgaris* Hildenborough was exposed to O_2_-driven laboratory adaptive evolution and acquired via point mutations as well as deletions/insertions the ability to gain energy from oxygen respiration under microoxic conditions (0.65% O_2_, Schoeffler et al. 2019). Since the enzymatic systems required for both sulfate and oxygen respiration were already present in the genome of *D. vulgaris*, only a limited number of mutations were apparently required to redirect the flow of reducing equivalents towards aerobic respiration coupled to growth (Schoeffler et al. 2019).

Marine *Acidobacteriota* were first indicated in *dsrAB* gene-based surveys by Müller et al. 2015 and recently their respective genomes could be recovered from marine surface sediments in the Arctic off the coast of Svalbard (Flieder et al. 2021). Here, they comprised the second most abundant DsrAB-encoding phylum after the *Desulfobacterota* (on average 13%) and represented 4% of *dsrB* transcripts, emphasizing their *in situ* activity. When expanded to a global marine *dsrAB* gene dataset, acidobacterial *dsrAB* genes averaged 15% in marine sediments worldwide (Flieder et al. 2021). They are affiliated to a different class (*Thermoanaerobaculia;* family FEB-10) than peatland Dsr-pathway encoding *Acidobacteriota* and comprise former uncultured *dsrAB* family-level lineage 9. Detailed annotation of their genomes revealed the metabolic potential for various respiratory pathways based on oxygen, nitrous oxide, metal-oxide, tetrathionate, sulfur and sulfate/sulfite as terminal electron acceptor. Potential electron donors comprised cellulose, proteins, cyanophycin, hydrogen, and acetate (Flieder et al. 2021). In summary, both terrestrial and marine Dsr-pathway encoding *Acidobacteriota* likely represent an ecologically important but so far overlooked group of SRM with a large metabolic versatility in respect to potential substrates including organic polymers and alternative electron acceptors including oxygen.

The metabolic flexibility to switch between sulfate reduction and aerobic respiration was also indicated in metagenomic and metatranscriptomic surveys of microbial mat-inhabiting members of the *Bacteroidota* family UBA2268 (Kapabacteria). In contrast to their well-studied phototrophic and sulfur-oxidizing relatives within the *Chlorobiaceae*, UBA2268-related MAGs retrieved from microbial mats of hot springs or groundwater encode reductive DsrAB as well as DsrD and DsrL-2C. For one of these MAGs (*Candidatus* Thermonerobacter thiotrophicus), the genome was annotated in greater detail and its transcriptional profile characterized during the diel cycle in the microbial mat of the thermal outflow of Mushroom Spring in Yellowstone National Park, USA (Thiel et al. 2019). Despite being a low-sulfate environment (<200 µM), the phototrophic microbial mat was characterized by high sulfate reduction rates (>5 µmol cm^−3^ d^−1^) during the night, which cease during daytime because of oxygen production by cyanobacteria-driven photosynthesis (Dillon et al. 2007). Accordingly, *Ca*. T. thiotrophicus showed strong expression of all Dsr-pathway genes during the night and a sharp decrease in its transcript levels during daytime. *Ca*. T. thiotrophicus also encoded a full respiratory chain including alternative complex III, an *aa*_3_-type low-affinity terminal oxidase as well as a *bd*-type high-affinity terminal oxidase. Interestingly, genes encoding the *aa*_3_-type low-affinity terminal oxidase were differentially expressed as compared to Dsr-pathway genes. Their highest expression levels were observed at light-dark transitions in the morning and evening (Thiel et al. 2019) corresponding to increasing and decreasing oxygen levels in the mat, respectively, but avoiding times of oxygen (over)saturation during daytime (Dillon et al. 2007), when genes encoding oxidative stress response dominated the transcriptional profile (Thiel et al. 2019). In contrast, genes encoding the *bd*-type terminal oxidase showed highest expression during the night implying a role in oxygen detoxification at low oxygen levels during active sulfate reduction. The absence of encoded CO_2_-fixation pathways and the increased expression of genes involved in glycolysis/gluconeogenesis, the TCA cycle, and acetate-related metabolism during the night indicated a heterotrophic lifestyle based on small organic molecules, which primarily correlated with sulfate reduction (Thiel et al. 2019).

Another intriguing group are the many DsrAB-encoding *Nitrospirota* members with an indicated reductive sulfur metabolism, which were encountered in environments of mainly moderate temperatures and that are distinct from their thermophilic, sulfate-reducing relatives within the genus *Thermodesulfovibrio*. When excluding *Thermodesulfovibrio* spp., 37 additional MAGs encoding an indicated reductively operating Dsr-pathway were recovered representing 10 candidate families within the phylum *Nitrospirota*. Typically, these MAGs were recovered from low-sulfate environments encompassing rice paddy soil (Zecchin et al. 2018), permafrost soils (Woodcroft et al. 2018), freshwater sediments, aquifer sediments, groundwater, the terrestrial and marine deep subsurface (Jungbluth et al. 2017, Anantharaman et al. 2018, Probst et al. 2018). However, a few were also recovered from brackish (Arshad et al. 2017) and saline marine environments (Kato et al. 2018). Among the mesophilic, low-sulfate adapted *Nitrospirota*, representatives from rice paddies were studied in more detail. From paddy soil that was used to grow rice in the presence and absence of gypsum (CaSO_4_ ·2 H_2_O), the partial genome of Dsr-pathway encoding *Candidatus* Sulfobium mesophilum (*Nitrospirota* family UBA6898) could be recovered. Parallel metaproteomics revealed active expression of its Dsr-pathway under gypsum amendment in support of a sulfate-reducing lifestyle. Interestingly, *Ca*. S. mesophilum also encoded the full pathway of dissimilatory nitrate reduction to ammonia, which was expressed in the treatment without gypsum amendment. The relative abundance of *Ca*. S. mesophilum was similar under both treatments, indicating that it maintains a stable population in rice paddy soils while shifting its primary energy metabolism. In contrast to the *Acidobacteriota* described above, *Ca*. S. mesophilum was rather adapted to the breakdown of classical substrates of SRM covering the metabolic potential to utilize butyrate, formate, H_2_, and acetate as an electron donor (Zecchin et al. 2018).

The *Actinomycetota* represented yet another unusual phylum harboring Dsr-pathwayencoding members (Müller et al. 2015). Besides the unusual *Actinomycetota* MAG GCA_003 599 855, which encoded reductive and oxidative DsrAB, all other retrieved *Actinomycetota* could be split into two major groups based on the completeness of their Dsr-pathway and habitat preference. All members of the class *Coriobacteriia* (five genomes/MAGs within the genera *Gordonibacter, Rubneribacter, Berryella*, and UBA8131) encoded only the genetic potential to reduce sulfite to sulfide including DsrD, lacked the *dsrL* gene, and were so far isolated or encountered in intestines of humans and animals including pig, chicken, and termites (Würdemann et al. 2009, Selma et al. 2014, Medvecky et al. 2018, Parks et al. 2018, Wylensek et al. 2020). Cultured representatives from the genera *Gordonibacter* and *Berryella* are strict anaerobes, supporting the notion that the encoded reductive bacterial-type DsrAB and DsrD point towards a reductive sulfur metabolism. However, dissimilatory sulfite reduction by these microorganisms still awaits experimental validation. Sulfite in the gut environment is likely derived from sulfonates, i.e. organic sulfur compounds with a SO_3_^2−^ moiety, such as the amino acid taurine (Wei and Zhang 2021) or the sugar sulfoquinovose (Hanson et al. 2021). The second major group within the Dsr-pathway encoding *Actinomycetota* encodes the full Dsr-pathway including DsrD and DsrL-2C. They represent uncultured members of the classes *Thermoleophilia* (Kato et al. 2018) and *Aquicultoria* (Jiao et al. 2021), with the latter encoding the oxygen-sensitive Wood-Ljungdahl pathway pointing towards a strictly anaerobic lifestyle and a reductively operating sulfur metabolism. In contrast to the intestinal and incomplete Dsr-pathway encoding members of the *Coriobacteriia*, they were found in terrestrial and marine environments including groundwater, the terrestrial subsurface (Jiao et al. 2021), and deep-sea massive sulfide deposits (Kato et al. 2018).

## Conclusion

Metagenome-driven discoveries have opened a new window into the hidden diversity of SRM. We can now start to appreciate that besides the four bacterial and two archaeal phyla harboring cultured SRM, the potential to perform dissimilatory sulfate/sulfite reduction extends to a total of 23 bacterial and 4 archaeal phyla. Many of the phyla now recognized to play a role in sulfur cycling were represented by DsrAB-encoding MAGs recovered from low-sulfate environments, supporting the notion that hidden or cryptic sulfur cycling in low-sulfate environments is an understudied area. For a few of these potential SRM, such as members of the *Acidobacteriota*, mesophilic *Nitrospirota*, and *Bacteriodata* family UBA2268 (Kapabacteria), meta-omics based studies under constrained environmental conditions could provide strong evidence of a sulfate-reducing lifestyle. However, the large majority of novel, putative SRM still await experimental confirmation of their physiology. Furthermore, we could show that the primers used in *dsrAB* gene-based approaches cover a large fraction of the novel diversity of SRM, with many of the previously taxonomically unresolved DsrAB lineages now anchored by DsrAB-encoding MAGs. As such, *dsrAB* gene-based surveys can be used with confidence in the future to explore the enigmatic world of a functional microbial guild that has shaped biogeochemical cycling on Earth since the Archaean (Shen et al. 2001, Wacey et al. 2011).

## Supplementary Material

fuad058_Supplemental_File
